# Experimental Study on the Compaction Characteristics and Evaluation Method of Coarse-Grained Materials for Subgrade

**DOI:** 10.3390/ma14226972

**Published:** 2021-11-18

**Authors:** Shanzhen Li, Yangsheng Ye, Liang Tang, Degou Cai, Shuang Tian, Xianzhang Ling

**Affiliations:** 1 School of Civil Engineering, Harbin Institute of Technology, Harbin 150090, China; lsz_hit@163.com (S.L.); ts_hit@163.com (S.T.); 2 Railway Engineering Research Institute, China Academy of Rails Science, Beijing 100081, China; yyshrails@163.com (Y.Y.); caidegou@126.com (D.C.); lingxianzhang@hit.edu.cn (X.L.)

**Keywords:** coarse-grained materials, compaction characteristic, subgrade, *CMV*, quality evaluation, geostatistical analysis

## Abstract

Coarse-grained materials are widely used in high-speed railway construction, and it is of great significance to research its compaction characteristics due to the high quality control requirements. In this regard, a field compaction experiment was conducted at a subgrade near Bazhou Station of Beijing-Xiong’an Intercity Railway. The test results of the compaction effect were presented in this study at first. The roller-integrated compaction measurements (i.e., compaction meter value, *CMV*) were compared with several traditional in-situ tests (i.e., plate load test, light falling weight deflectometer test, and shear wave velocity test). Then the stability of *CMV* was evaluated by the proposed *δ* criterion. The spatial uniformity of compaction was further investigated. Based on the analysis, the target value of *CMV* was preliminarily determined. It showed that *E*_vd_ was more variable than *CMV*. The results convincingly indicated that the compaction parameters increased with the increasing number of roller passes at first. A further increase in compaction effort could result in the decompaction of material when the compaction number up to a certain value. The stability analysis method proposed in this study showed its potency of quantifying the percentage of areas with acceptable compaction. The geostatistical analysis could reflect the spatial uniformity of compaction. Overall, the conducted study could provide a useful reference for geo-material compaction control in the transportation engineering.

## 1. Introduction

Coarse-grained materials are widely used in high-speed railway construction due to its good compaction performance and high shear strength. Compaction is one of the important aspects of subgrade construction [1,2], and it determines the service performance of high-speed railway [3,4].

Compactness of subgrade soils is commonly evaluated by various traditional indexes, such as degree of compaction (*K*), the modulus of subgrade reaction (*K*_30_), dynamic deformation modulus (*E*_vd_), and deformation modulus (*E*_v2_). According to previous research findings, the traditional compaction indexes exist several shortages. First, using limited sample values (the sampling area is less than 1%) cannot reflect the compaction quality of the entire compaction area, which may cause test biases [5]. Second, failing to obtain compaction information synchronously may result in insufficient or excessive compaction [6,7]. Third, damages to the compacted layer, long test time, and heavy equipment loads cause significant interference to the subsequent construction process [7]. Forth, control of the compaction process through artificial supervision is prone to unreliable results [5]. Finally, reworking after problems may delay the construction period [8,9].

In recent years, continuous compaction control (CCC) has been becoming the standard technology for testing the compaction performance of vibratory rollers [10,11,12]. Compared with the traditional subgrade compaction quality control practice, the CCC quality evaluation has significant advantages [13]. Vennapusa et al. [14] collectively referred to various measurement values as roller-integrated compaction monitoring, which mainly contains the compaction meter value (*CMV*) [15], the compaction control value (*CCV*) [16], the machine drive power (*MDP*) [17], and the stiffness index (*K*_s_) [13]. Moreover, many researchers have focused on the relationship between continuous compaction indexes and traditionally-used measurements [5,18,19,20,21]. The key findings have been summarized by Cai et al. [22]. White and Thompson [23], for instance, have revealed a strong linear correlation between the roller and in-situ measurements using statistical averaging analysis.

Previous studies also have found that continuous compaction can significantly improve compaction efficiency and uniformity, achieving full coverage of the compaction area monitoring [14,18]. There are also many studies that used geostatistical analysis to evaluate the compaction uniformity, all of which have achieved good results [23,24,25]. Grabe [26] evaluated the spatial variation of soil stiffness by analyzing the spectral density of the measured value of the roller. Facas et al. [27] further measured the anisotropy of soil stiffness in spatial distribution. In addition, a large number of studies have also shown that continuous compaction can effectively identify weak areas of the compacted layer, avoiding insufficient or excessive compaction [25].

To research the compaction characteristics of coarse-grained materials, a series of tests were carried out at the subgrade of Beijing-Xiong’an Intercity Railway. The relationships between traditional in-situ measurements and roller measurements were analyzed by statistical analysis. Then a more applicable criterion was established to evaluate the stability of compaction effect. The spatial uniformity of compactness was further investigated using the semivariogram model.

## 2. Field Compaction Experiment

### 2.1. Test Materials

**Figure 1:**
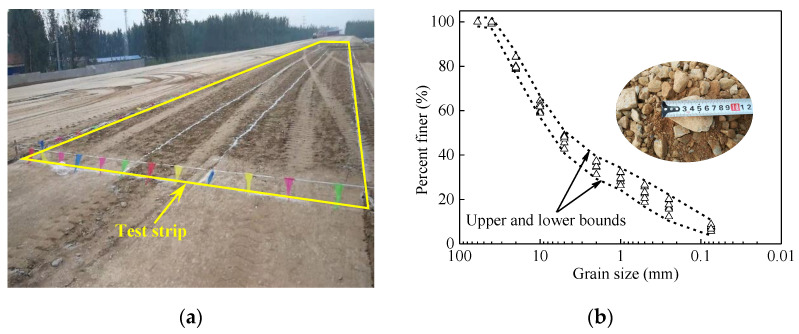
Layout and material for the experimental field test. (**a**) view of test strip; (**b**) grain size distribution.

The field test area is close to Bazhou Station of Beijing-Xiong’an Intercity Railway. The layout of the experimental field is shown in Figure 1a. The filling material is coarse-grained with the maximum dry density of 2.27 g/m^3^ and the optimal moisture content of 5.2%. The grain size distribution curve is shown in Figure 1b. The average uniformity coefficient (*C*_u_) and the average curve coefficient (*C*_c_) are 77.82 and 1.15, respectively. The testing plan was designed with a length of 80 m. The thickness of the granular material was about 0.3 m for loose.

The filling materials were loosely spread over the compacted subgrade. Then a single static and nine weak vibration compactions were carried out. According to the field test, the average elevations of soil surface before and after the static compaction were about 14.256 m and 14.250 m, respectively. The dry density and water content measured after the static compaction were controlled at 1.97 g/cm^3^ and 5.4%. The vibrating roller performed in weak vibration mode at a speed of 3 km/h. The duration time for each compaction was about 2 min. After each compaction, the in-situ tests were carried. The interval between each vibration compaction was about an hour.

### 2.2. Measurement of Compaction Degree

**Figure 2:**
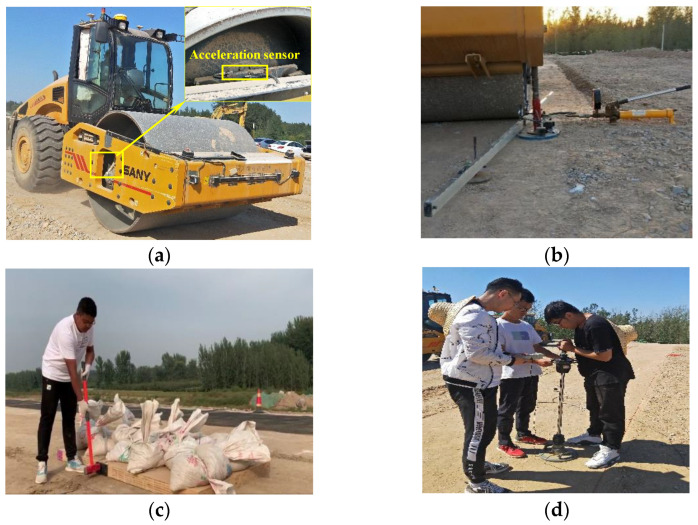
Field tests. (**a**) vibratory roller drum; (**b**) plate load test (*K*_30_); (**c**) shear-wave velocity test; (**d**) light falling weight deflectometer (LWD) test.

As shown in Figure 2a, the field compactions were carried out with a single-wheel vibratory roller (SSR260C-6), which was produced by San–Heavy industry Co., Ltd. in Beijing, China. The total weight of this compaction roller is 26.7 t. The roller has a drum with diameter of 1.70 m and width of 2.17 m. The power of the vibration roller is 180 kW, which can provide a vibration frequency of 27–31 Hz and a vibration amplitude of 1.03–2.05 mm. The spatial coordinates can be obtained by the global positioning system (GPS).

**Figure 3:**
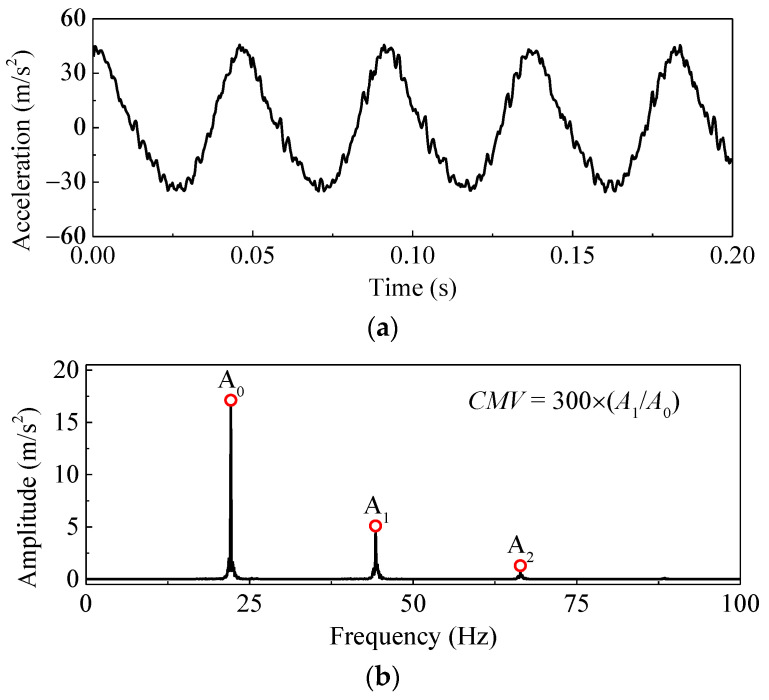
Method to determine *CMV* (pass 1, for example). (**a**) acceleration time history of roller drum; (**b**) acceleration frequency spectra of roller drum.

*CMV* technology, which has a clear physical background, indicates the interaction between vibratory drum and subgrade soil stiffness [5]. The dynamic response signal was collected by the accelerometer installed on one side of the vibration roller as shown in Figure 2a. The accelerometer was located at the junction point of the vibration roller’s longitudinal and vertical axes, so that the response of accelerometer could reflect the vibration of roller. As shown in Figure 3, the Fast Fourier Transform (FFT) analysis technique is applied to the dynamic signal for spectral analysis. In addition to the fundamental frequency signal, the frequency spectrogram also contains signals of other frequency components. The *CMV* is defined as the amplitude ratio of the first harmonic frequency and the fundamental frequency [8,15], which can be calculated as:

(1) $$CMV=c\frac{A_{1}}{A_{0}}$$

where *c* is a constant value (normally about 300); *A*_0_ and *A*_1_ are the amplitudes of the fundamental component and first harmonic component of the vibration, respectively.

To determine the relationship between roller and in situ compaction measurement parameters, it is necessary to conduct a series of in-situ tests on the subgrade soil. The in-situ compaction measurement parameters mainly include the modulus of subgrade reaction (*K*_30_) [28], the dynamic deflection modulus (*E*_vd_) [29], and the shear wave velocity (*V*_s_) [30,31]. They are determined by plate load test, light falling weight deflectometer (LWD) test and shear wave velocity test, respectively, as shown in Figure 2.

The dynamic deformation modulus (*E*_vd_) is an index reflecting the ability of soil to resist deformation under a vertical impact force. It can be calculated according to Equation (2).

(2) $$E_{\mathrm{vd}}=\frac{2\left(1-\nu^{2}\right)}{\pi \cdot r}\cdot \frac{Q_{\max}}{s}$$

where *v* is Poisson’s ratio of soil; *r* is the radius of plate; *Q*_max_ is the maximum dynamic impact load; and *s* is vertical deflection of the soil surface.

**Figure 4:**
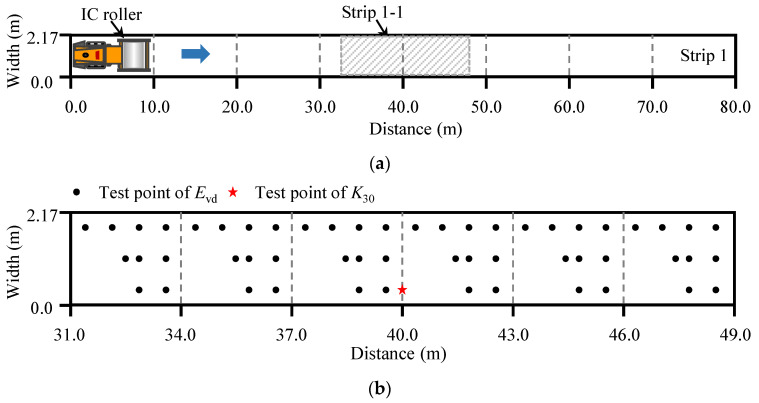
Testing plan. (**a**) strip 1; (**b**) strip 1-1.

The test points for determining *E*_vd_ and *K*_30_ are shown in Figure 4. To consider the spatial uniformity of compaction, 54 different test points for *E*_vd_ were laid out except for the areas on the south-western segments. The *E*_vd_ were tested after each roller pass. Other tests, such as the test of water content and the ground elevation surveying, were conducted on the south-western regions. The *V*_s_ was measured in the entire test site. After the 1st, 3rd, 5th, and 7th roller passes, the plate load test was conducted to obtain *K*_30_. The density and water content were measured by sand filling method after passes 3, 6 and 9.

## 3. Results and Analyses

### 3.1. Practical Observations

**Figure 5:**
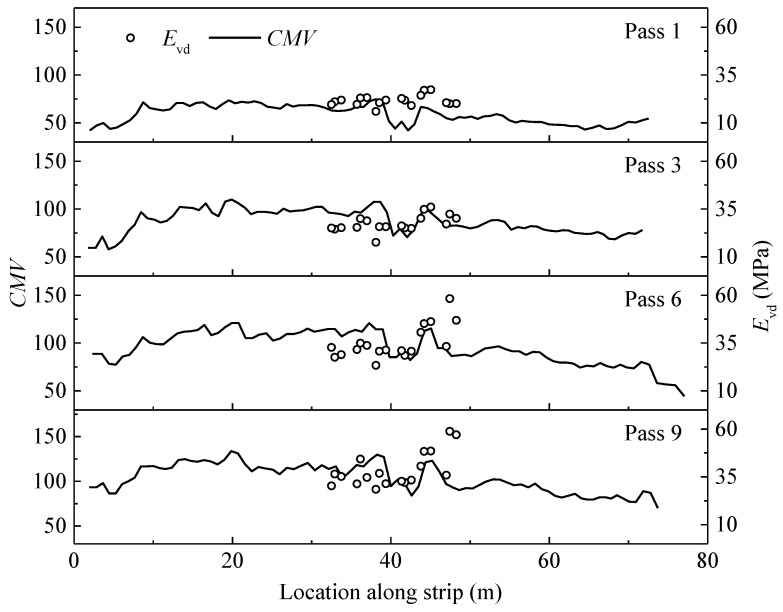
Variation of *CMV* and *E*_vd_ after different passes.

*CMV* and *E*_vd_ were obtained along the entire length of the test strip (Figure 5). *CMV* measurements are represented with solid lines, and *E*_vd_ results are shown as discrete points. The similar changing rules of *CMV* and *E*_vd_ indicate that *CMV* has a certain correlation with the stiffness of subgrade materials. It should be noted that the trend of *CMV* is not completely coincident with *E*_vd_. The reason for this is that the influence depths of the two measurement methods are different [8,32].

**Figure 6:**
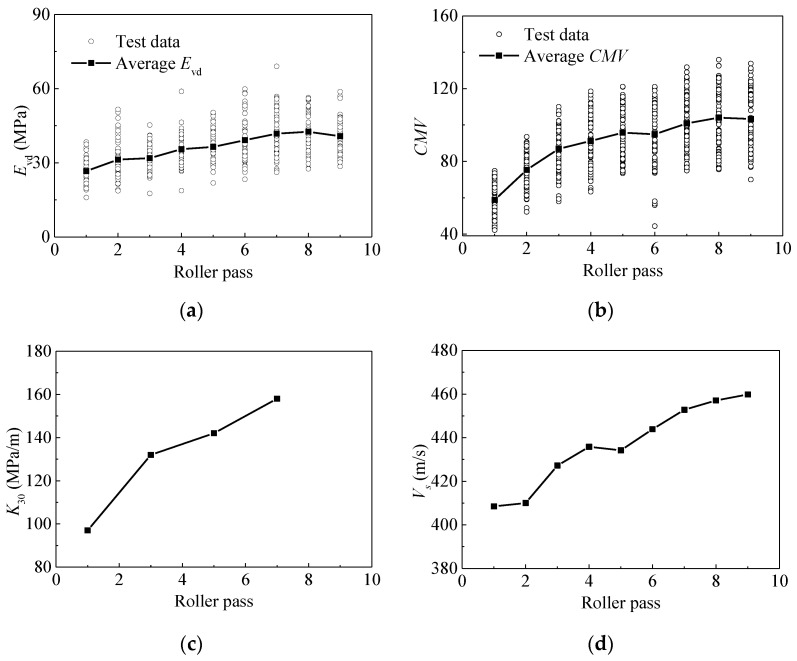
Compaction curves after different compaction pass. (**a**) *E*_vd_; (**b**) *CMV*; (**c**) *K*_30_; (**d**) *V*_s_.

**Figure 7:**
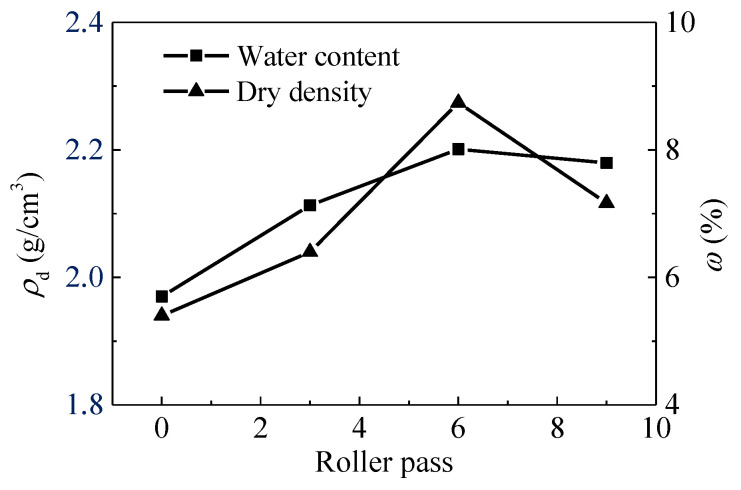
Curves of dry density (*ρ*_d_), and water content (*ω*) after different compaction pass.

Figure 6 demonstrates the changing pattern of *E*_vd_, *CMV*, *K*_30_, and *V*_s_ after each compaction pass. It can be observed that these indices show a clear increasing trend, despite certain scattering, which can roughly reflect the relationship between test indices and compaction state. The averaged *E*_vd_ and *CMV* increase from approximately 26.64 MPa and 58.80 to 40.74 MPa and 103.28, respectively. After eight passes, slight decreases in *E*_vd_ and *CMV* are observed. The reduction in *E*_vd_ and *CMV* could be due to the loosening of soils. The response is further supported by the result of dry density. As shown in Figure 7, the dry density of pass 9 is less than that of pass 6. The test result of water content is also obtained. It shows that the water content keeps changing during different passes due to complex factors, such as the change of weather conditions, the moisture migration in soils and the condensation of moisture in the air. The water content may affect the compaction quality. The comparison of *CMV* and *E*_vd_ shows that *E*_vd_ is more variable than *CMV*. This can be attributed to the fact that manual measurements may produce unreliable results.

### 3.2. Statistical Analysis of Test Indexes

**Figure 8:**
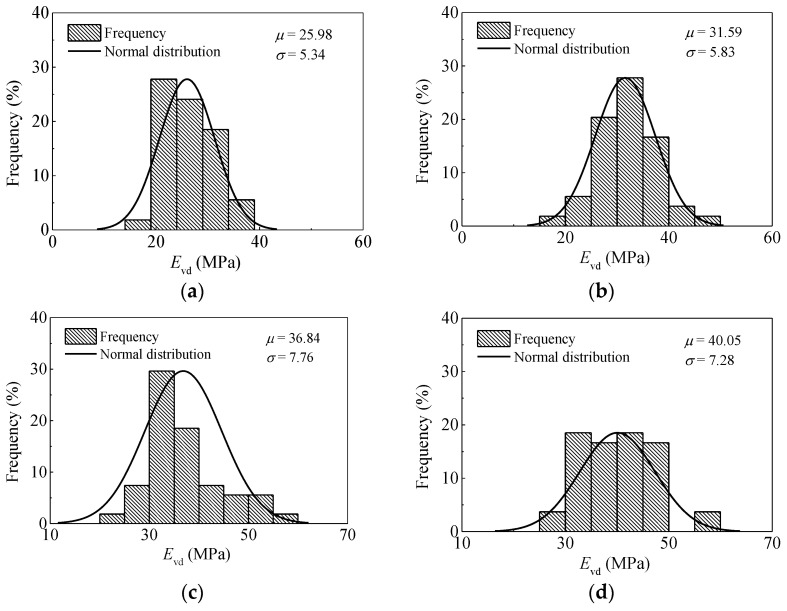
Variation and distribution of the *E*_vd_ after different passes. (**a**) pass 1; (**b**) pass 3; (**c**) pass 6; (**d**) pass 9.

The univariate statistics (i.e., mean *μ* and standard deviation *σ*) are typically used as compaction effect control criteria. The histograms and distribution curves of *E*_vd_ for passes 1, 3, 6, and 9, are given in Figure 8. This information can help in the determination of compaction degree. The range of *E*_vd_ after the first compaction is between 15.95 MPa and 38.40 MPa. The average value of *E*_vd_ is about 25.98 MPa with a standard deviation of 5.34 MPa. The coefficient of variation (*COV*) is 20.55%, demonstrating that the section cannot be strictly considered uniform. The range of *E*_vd_ after the ninth compaction is shown as a histogram in Figure 8d, which is between 28.52 MPa and 58.75 MPa. The average value is about 40.05 MPa with a standard deviation of 7.28 MPa (*COV* of 18.18%). Actually, these wide variations highly influence the relationships between different parameters and compaction quality [33].

**Figure 9:**
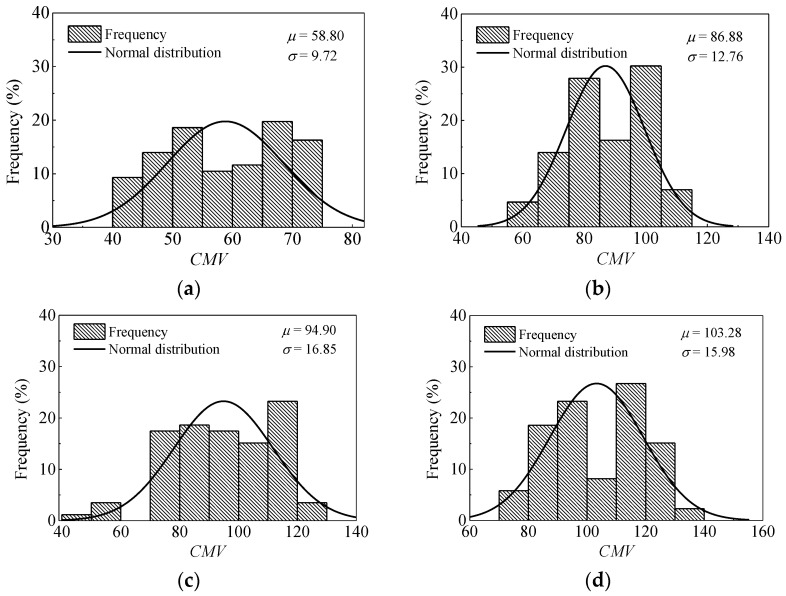
Variation and distribution of the *CMV* after different passes. (**a**) pass 1; (**b**) pass 3; (**c**) pass 6; (**d**) pass 9.

Figure 9 illustrates the histograms and distribution curves of CMV for passes 1, 3, 6, and 9. According to Figure 5, *CMV* along the entire length of the test strip is fluctuant, and it increases at first and then decreases as a whole. The non-uniformity of the subgrade may be attributed to the heterogeneity and moisture content variations of coarse-grained materials. It can be seen from Figure 9 that there exist two distinct peaks in most of the datasets. This is possibly be caused by the non-uniformity of the subgrade fillings and the use of the statistical interval. In general, the trend of *CMV* is the similar to *E*_vd_. The parameters of statistical analysis on *E*_vd_ and *CMV* are summarized in Table 1. With the increase of roller passes from 1 to 8, the mean *E*_vd_ rises from 25.98 MPa to 41.73 MPa, and the coefficient of variation changes in the range from 17.75% to 22.19%. For *CMV*, the mean value rises from 58.80 to 104.04. No definite trend in *COV* is observed. The values fluctuate in the range from 12.68% to 17.76%. With a further increase in roller pass (i.e., pass 9), both the mean *E*_vd_ and *CMV* decrease slightly. This phenomenon could be due to the loosening of soils, indicating that there is no additional need for compaction.

### 3.3. Relationships between Measured Parameters

**Figure 10:**
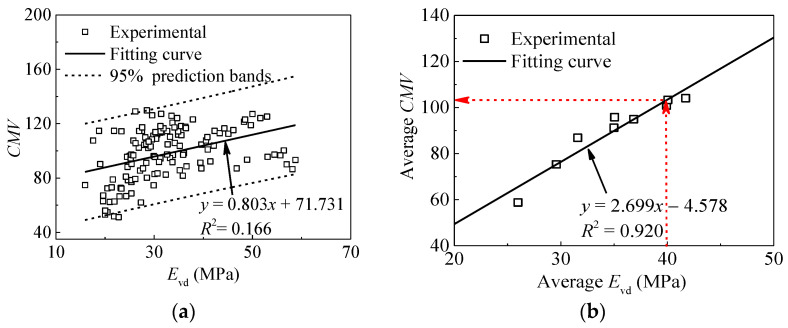
Relationships between *E*_vd_ and *CMV*. (**a**) statistical analysis; (**b**) statistical averaging analysis.

As mentioned above, *CMV* and *E*_vd_ are both capable of reflecting the compaction effect. Figure 10a presents the relationship between *CMV* and *E*_vd_ obtained in Strip 1. It can be seen that the test data are not strongly correlated. The main reasons for this difference can be attributed to that: (a) the sampling area of LWD test is less than that of roller measurements; (b) the heterogeneity and moisture content of subgrade materials are variable; and (c) the influence depths of the two measurements are different. As reported in references [8,32], the influence depth of rollers is about 0.5 m–1.5 m, which is much deeper than that of the LWD test (about 0.2 m). This is an important reason for the poor correlations between *CMV* and *E*_vd_.

Considering the variability of different methods, the statistical regression analysis is performed. The coefficient of determination (*R*^2^) is adopted to evaluate the reliability of the model. In general, the coefficient of the regression equation obtained with grouping data is better than that without grouping data. Here, the data of *CMV* and *E*_vd_ are grouped by the compaction number. Therefore, the results are averaged to produce a single data point for each roller pass. The statistical averaging of the data clearly mitigates the measurement variation and position error, and reveals underlying trends [33]. Figure 10b presents the simple linear relationships between averaged *CMV* and *E*_vd_. The coefficient of determination (*R*^2^) is 0.908, revealing statistically significant relationships between in-situ and roller measurements. The criterion for acceptance in the production area is that the minimum *E*_vd_ is 40 MPa for base course materials according to the Code for Design of High Speed Railway [34]. Based on the regression formula and the minimum *E*_vd_ required by the specification, the target *CMV* value (i.e., 103) can be back-calculated. This value can be used as a reference for compaction degree control in the production areas.

**Figure 11:**
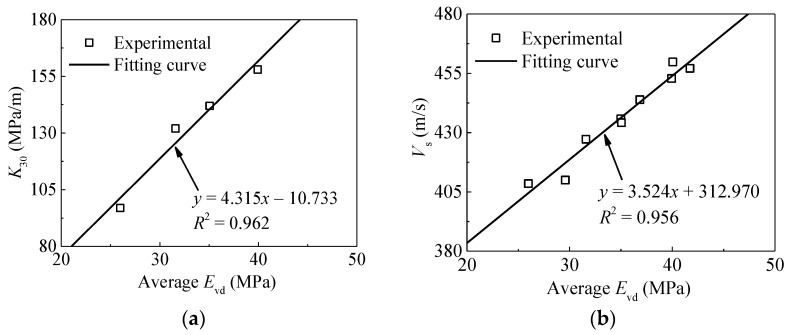
Relationships between *K*_30_, *V*_s_ and average *E*_vd_. (**a**) *K*_30_ versus average *E*_vd_; (**b**) *V*_s_ versus average *E*_vd_.

**Figure 12:**
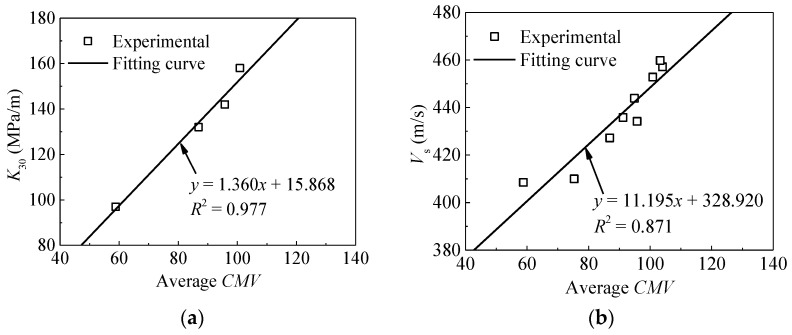
Relationships between *K*_30_, *V*_s_ and average *CMV*. (**a**) *K*_30_ versus average *CMV*; (**b**) *V*_s_ versus average *CMV*.

The relationships between averaged *E*_vd_ and other in-situ compaction measurements (*K*_30_ and *V*_s_) are also depicted in Figure 11. The results indicate relatively clear and strong linear trends with *R*^2^ values both exceeded 0.85. As shown in Figure 12, the same relationship is observed for *CMV*. This means that statistical averaging method could mitigate measurement variability and reveal underlying trends. Considering the conventional methods are time-consuming and destructive, therefore they are unable to meet the requirements of rapid construction [7]. Consequently, only *CMV* is analyzed in the following section.

### 3.4. Compaction Stability

**Figure 13:**
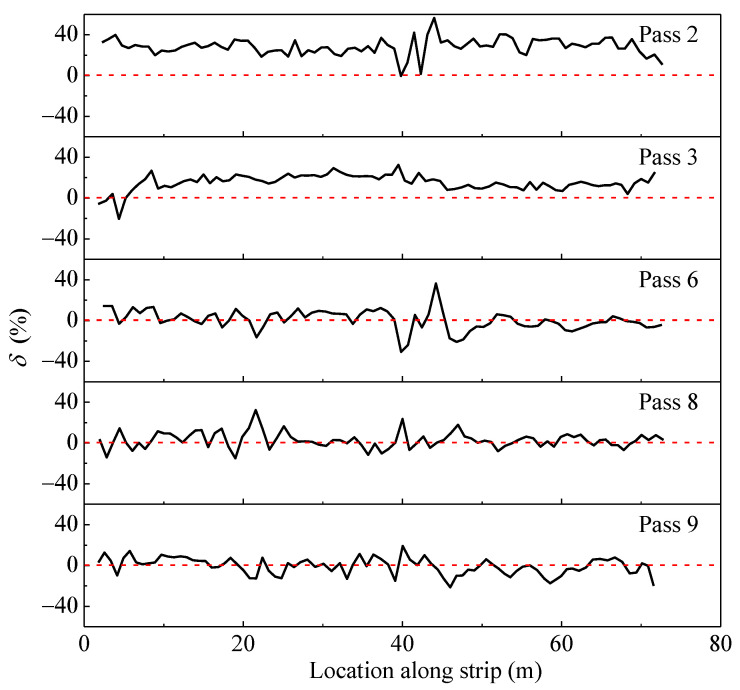
Variation of *δ* after different passes.

The variation of point-by-point differences between the values of *CMV* (Δ*CMV*) before and after each compaction is included in Figure 13. A positive Δ*CMV* indicates that the stiffness has been improved, which can be translated into an effective compaction effort. While the negative Δ*CMV* reflects that the area has not been compacted effectively. To reflect the change degree before and after each compaction, a simple parameter is defined as follows:

(3) $$\delta =\frac{CMV_{n}-CMV_{n-1}}{{\bar{CMV}}_{n-1}}\times 100\%,\left(n\geq 2\right)$$

where *CMV_n_* is the value of *CMV* at different locations after pass *n*; *CMV_n_*_−1_ is the value of *CMV* at different locations after pass *n* − 1; ${\bar{CMV}}_{n-1}$ is the average of Δ*CMV* after pass *n* − 1.

As shown in Figure 13, when the compaction number is lower (such as pass 3), the values of *δ* in the most section are above zero lines, which means the material was efficiently compacted. With the increase of compaction numbers (such as passes 6, 8, and 9), the data fluctuate within a certain range around zero lines. It should be noticed that most of the values are negative from 45 m to 70 m along the strip, especially for passes 6 and 9, indicating the decompaction of material.

**Figure 14:**
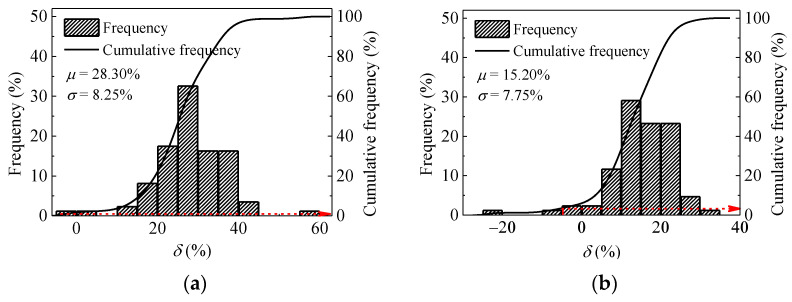

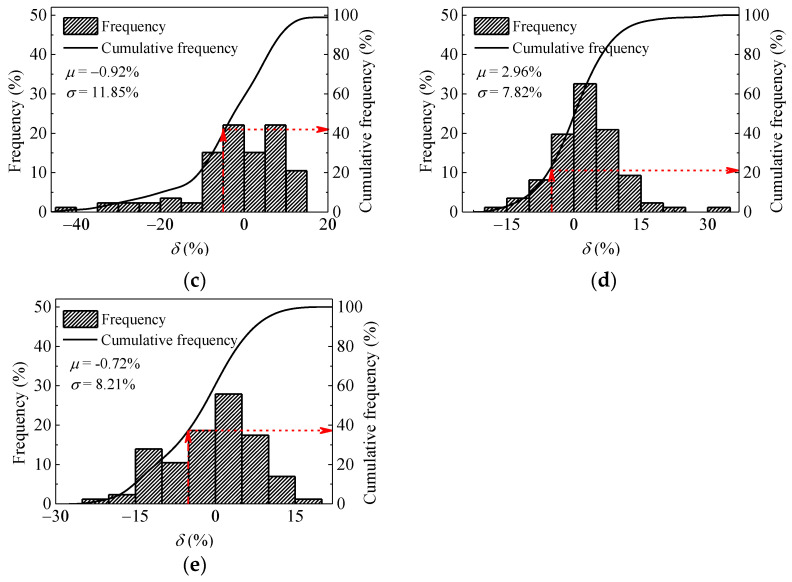
Variation and distribution of *δ* after different passes. (**a**) pass 2; (**b**) pass 3; (**c**) pass 6; (**d**) pass 8; (**e**) pass 9.

The coordinates of roller drum can be obtained by GPS. The value of *CMV* corresponding to the coordinates can also be recorded by the roller measurement. Small uncertainties in the GPS coordinates may negatively impact the relationship between *CMV* and locations [35]. To attenuate the uncertainty in the *CMV* measurements and locations, an acceptable value is defined as −5%. Frequency distribution plots for *δ* after passes 3, 6, 8, and 9 are presented in Figure 14. To quantify the percentage of areas with acceptable compaction, the distribution can be translated to cumulative distribution. When the compaction number is lower (such as passes 2 and 3), the frequency distribution is skewed to the left, which means most of the measurement points are effectively compacted. The percentages of compacted areas after passes 2 and 3 are 100% and 97.7%, respectively. With the growth of passes (such as pass 8), *δ* tends to be a normal distribution (as illustrated in Figure 14c) and the mean value of *δ* is larger than zero. About 78.58% of the section area is compacted effectively. However, when the pass further increases to 9, the mean value of *δ* is less than zero. This phenomenon reflects the decrease of stiffness, which is consistent with the previous finding obtained in Figure 6.

In this paper, the mean value of *δ* is defined as the process control index of compaction stability. It depends on the engineering importance rating, the compaction thickness, the total weight of rollers and so on. In general, the absolute value of this index is not larger than 5% according to the Code for Design of High-Speed Railway [34]. A summary of averages and standard deviations for *δ* are presented in Table 2. The average *δ* generally decreases with the increase roller passes. The standard deviation varies from 3.91% to 12.33%. In this study, 5% is selected as the control value of compaction stability. With this control value, the data of pass 8 (the mean value of *δ* is 2.96) shows greater stability than other roller passes, which would meet the requirement of stability. Based on the above results, the average value of *CMV* after pass 8 exceeds the target value of *CMV* (103). A total of 78.58% of the strip is compacted effectively. With these criteria, the optimum compaction number (pass 8) is obtained in this strip.

### 3.5. Spatial Uniformity of Compaction

For an ideal compacted region, an equally important issue is to keep the compaction degree uniform [35]. The univariate statistics analysis mentioned in last section cannot reflect the spatial uniformity of compaction. Therefore, the results are further investigated using the geostatistical analysis to evaluate compaction quality.

Here, the semivariogram model is used to quantify spatial uniformity [36]. The semivariogram *γ*(*h*) is computed as follows:

(4) $$\gamma (h)=\frac{1}{2n(h)}{\sum_{i=1}^{n(h)}\left[z(x_{i}+h)-z(x_{i})\right]}^{2}$$

where *z*(*x_i_*) is the measurement taken at location *x_i_*; *n*(*h*) is the number of data pairs *h* units apart in the research direction; *h* is the lag.

To estimate the semivariogram properties, an exponential model is selected to fit the experimental semivariogram in this study.

(5) $$\gamma (h)=C_{0}+C\left(1-e^{-h/a}\right)$$

where *C*_0_ is nugget; *C* is the partial sill; *C* + *C*_0_ is named sill; *a* is the range. It should be noted that *C*_0_ reflects the measurement error at the origin of the semivariogram; *C* + *C*_0_ means the variance of data; *a* is the distance for the semivariogram reaching the plateau.

**Figure 15:**
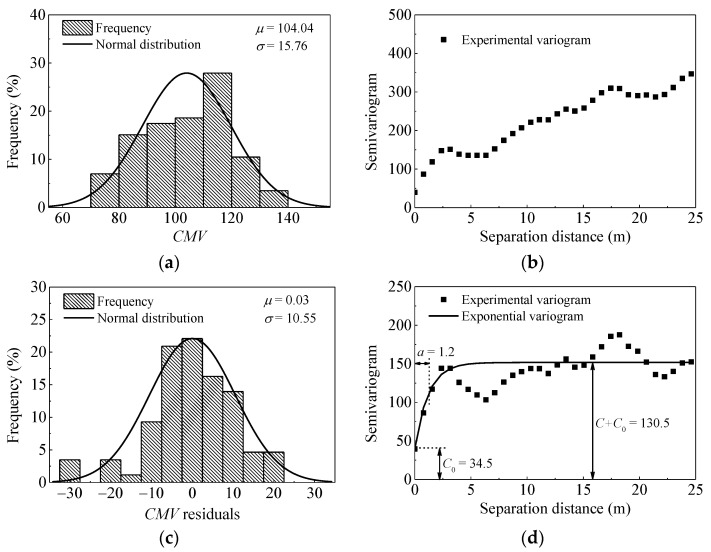
Histogram and semivariogram plots of *CMV* after pass 8. (**a**) distribution of *CMV*; (**b**) semivariogram of *CMV*; (**c**) distribution of *CMV* residuals; (**d**) semivariogram of *CMV* residuals.

The frequency distribution and semivariogram results for *CMV* after pass 8 are presented in Figure 15. It can be seen from Figure 15b that the semivariogram exhibits an erratic behavior. This means that as the distances increase, the differences between data also increase. In other words, the data shows a systematic trend. This trend must be removed before geostatistical analysis [37]. Similar to the approach presented in the reference [14], a quadratic model is used to remove the trend. Then, residual values of *CMV* show a clear spatial structure as shown in Figure 15d.

**Figure 16:**
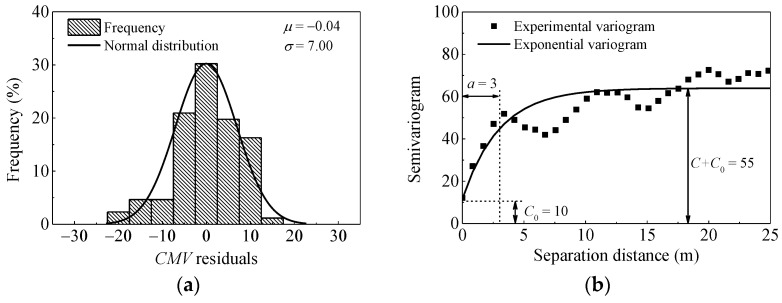
Histogram and semivariogram plots of *CMV* after pass 1. (**a**) distribution of *CMV* residuals; (**b**) semivariogram of *CMV* residuals.

In order to compare the evaluation index of spatial uniformity, results of pass 1 are plotted in Figure 16. As illustrated in Figure 15d and Figure 16b, the sill value of pass 1 (*C*_0_ = 10 and *C* = 45) is quite lower than that of pass 8 (*C*_0_ = 34.5 and *C* = 96.5), indicating less variability in pass 1. Larger range value is observed after pass 1 (*a* = 3 m) than that of pass 8 (*a* = 1.2 m), which demonstrates better spatial continuity at the start of the compaction.

**Figure 17:**
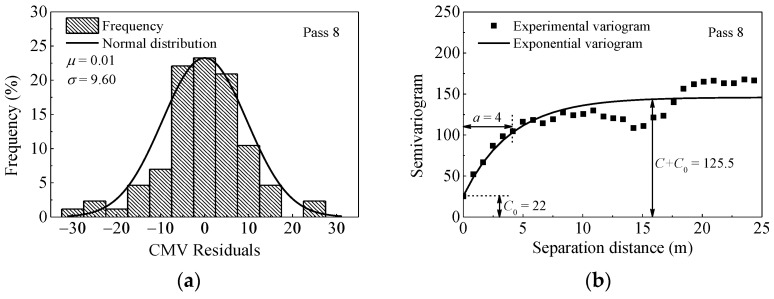
Comparison between calibration and proof areas with spatial statistics (strip 2). (**a**) distribution of *CMV* residuals; (**b**) semivariogram of *CMV* residuals.

The results of pass 8 from another strip (named Strip 2) are also listed for comparison. The test Strip 2 was intentionally prepared with no destructive tests to demonstrate the influence of such conditions on semivariogram modeling. The results are presented in Figure 17. As observed in Figure 15d and Figure 17b, the value of *C* + *C*_0_ in Strip 2 is lower than that of Strip 1, indicating less variability in Strip 2. The larger range value of Strip 2 (*a* = 4 m) also indicates longer spatial continuity. The comparison of these two calibration strips shows that there exit significant differences in their spatial statistics. This is expected as in-situ tests (such as sand filling method) conducted in Strip 1 are destructive, which influences the uniformity of materials. The geostatistical analysis provides parameters to quantify the spatial uniformity of compacted materials. This approach combined with the stability criteria can help to identify the regions of noncompliance.

## 4. Conclusions

The compaction characteristics of coarse-grained materials were investigated at the subgrade of Beijing-Xiong’an Intercity Railway by continuous compaction measurements and in-situ measurements. The relationships between *CMV* and in-situ measurements were analyzed using statistical analysis. Then the stability and the spatial uniformity of *CMV* were further investigated. The main conclusions gained from the above work can be drawn as follows:

(1) The compaction trend is similar for both the traditional in situ and continuous compaction measurements. When the compaction number is up to a certain value (8th compaction in this study), a further increase in compaction effort could result in the decompaction of material. The dynamic deformation modulus *E*_vd_ is observed to be more variable than *CMV*.
(2) The correlations between *CMV* and in-situ compaction measurements are strong and stable enough by using statistical averaging analysis. A regression formula between *CMV* and *E*_vd_ is established to determine the target value of *CMV*.
(3) The stability analysis proposed in this study will help to quantify the percentage of areas with acceptable compaction and identify the optimum compaction number. The geostatistical analysis reflects the spatial uniformity of compaction. Based on these two criteria, the optimum compaction number (pass 8) is obtained in strip 1.
(4) The stability analysis and spatial uniformity analysis could aid the contractor in identifying poorly compacted areas or areas with highly non-uniform conditions that need additional compaction or other modification. These methods can help to improve the quality of construction, enhance the performance of pavements, and reduce the cost of construction.

## Figures and Tables

**Table 1:** The summary of the statistical analysis results for compaction measurements.

Pass No.	*E*_vd_			*CMV*		
	Average Value (MPa)	Standard Deviation (MPa)	Variation Parameter *COV* (%)	Average Value	Standard Deviation	Variation Parameter *COV* (%)
1	25.98	5.34	20.55	58.80	9.72	16.53
2	29.58	6.10	20.62	75.42	9.56	12.68
3	31.59	5.83	18.46	86.88	12.76	14.69
4	34.99	6.97	19.92	91.18	14.60	16.01
5	35.05	6.22	17.75	95.78	13.21	13.79
6	36.84	7.76	21.06	94.90	16.85	17.76
7	39.93	8.86	22.19	100.88	14.97	14.84
8	41.73	7.87	18.86	104.04	15.76	15.15
9	40.05	7.28	18.18	103.28	15.98	15.47

**Table 2:** The statistic results of *δ* after different passes.

Pass No.	Mean Value, *μ* (%)	Standard Deviation, *σ* (%)	Acceptable Compaction Percentage (%)
2	28.30	8.25	100
3	15.20	7.75	97.68
4	4.52	6.72	86.46
5	4.40	3.91	89.58
6	−0.92	11.85	58.62
7	5.97	12.33	78.43
8	2.96	7.82	78.58
9	−0.72	8.21	62.52

## Data Availability

The data presented in this study are available on request from the corresponding author.
